# DEF-Net: A dual-modal feature enhancement and fusion network for infrared and visible object detection

**DOI:** 10.1371/journal.pone.0345815

**Published:** 2026-04-01

**Authors:** Xiaoming Guo, Fengbao Yang, Linna Ji

**Affiliations:** 1 Shanxi Key Laboratory of Machine Vision and Virtual Reality, North University of China, Taiyuan, China; 2 School of Information and Communication Engineering, North University of China, Taiyuan, China; PLOS, UNITED KINGDOM OF GREAT BRITAIN AND NORTHERN IRELAND

## Abstract

Infrared-visible object detection in complex dynamic environments often suffers from weak feature representation and underutilized cross-modal complementarity, leading to missed and false detections. To address these issues, we propose a Dual-modal Enhanced Feature Enhancement and Fusion Network (DEF-Net). To enhance the model’s focus on informative features within both infrared and visible modalities, a feature interaction enhancement module is designed to effectively highlight and reinforce salient information. Furthermore, to better exploit the complementary characteristics of the two modalities, a transformer-based fusion architecture incorporating a cross-attention mechanism is introduced, enabling deep inter-modal feature integration. Experiments on SYUGV and LLVIP datasets show that DEF-Net outperforms existing methods in accuracy while maintaining real-time processing speed.

## 1. Introduction

Unmanned mobile platforms are widely used in autonomous driving, surveillance, and disaster response due to their mobility, sensing capability, and low risk. These systems typically rely on target detection technologies to execute tasks [1]. However, in complex environments, images acquired by single-sensor systems are often degraded by various environmental factors, resulting in compromised recognition performance. To support applications across diverse scenarios, unmanned platforms are often equipped with dual-modal vision sensors, with infrared and visible sensors being among the most widely deployed for target detection [2]. Visible images offer rich texture and color details but are highly sensitive to changes in lighting and visibility. In contrast, infrared imagery, which captures thermal radiation information, remains relatively invariant to illumination variations, though it typically exhibits lower spatial detail and overall image quality [3]. By integrating visible and infrared sensors, the complementary characteristics of both modalities can be effectively leveraged to achieve more robust and accurate target detection under varying environmental conditions.

Pedestrian and vehicle detection using infrared-visible image pairs is a typical multimodal detection task. However, it faces challenges such as complex environments with blurred targets and the difficulty of effectively leveraging cross-modal complementary information [4]. To address these issues, several researchers have adopted fusion-based approaches that combine infrared and visible imagery, using the integrated result for subsequent target recognition. For instance, Yue et al. [5] employed a saliency detection network to generate target saliency maps from infrared images, followed by fusion with visible images via non-subsampled contourlet transform (NSCT). The combined saliency and fusion results were then fed into a detector, demonstrating improved accuracy under low-light conditions. Similarly, Liu et al. [6] introduced a Target-aware Dual Adversarial Learning (TarDAL) framework to merge infrared and visible images into a single representation, which was subsequently processed by a YOLOv5 detector. Tang et al. [7] developed a parameter transfer model and later proposed a deep learning-based decision-level fusion framework for joint infrared-visible detection. Sun et al. [8] designed a triple-branch recognition network named UA-CMDet, incorporating separate branches for visible, infrared, and fused features, with ResNet-50 serving as the backbone for feature extraction, followed by decision integration across branches. Despite these efforts, front-end fusion strategies tend to preserve both foreground and background details in the fused modality, often introducing redundant information that can hinder recognition performance. On the other hand, back-end fusion methods, which operate at the decision level, typically lack the ability to exploit cross-modal interactions during feature learning and often require multiple models, leading to high computational costs.

Feature-level fusion is a dominant approach in multimodal detection, effectively integrating complementary information while keeping the model lightweight. For instance, Geng et al. [9] developed a dual-branch detection network based on Faster R-CNN that processes paired visible and infrared images through a shared VGG-16 backbone for separate feature extraction, followed by the fusion of both branches via classification and bounding box regression modules. Xue et al. [10] introduced a real-time detection model named MAF-YOLO, which incorporates an image brightness attention mechanism. Their framework uses a Darknet-53-based multimodal feature extraction module and integrates the features through a modal-weighted fusion module. Cheng et al. [11] proposed a lightweight dual-modal fusion network called SLBAF-Net, which includes a Bimodal Adaptive Fusion Module (BAFM) to adaptively merge visible and infrared feature maps, thereby improving detection robustness in challenging environments. Recognizing that conventional CNNs are limited to local receptive fields, Shen et al. [12] designed a fusion method guided by bidirectional attention. This approach employs a Transformer architecture to enable global feature interaction and complementary information capture between visible and infrared modalities, enhancing the discriminability of target features and alleviating the issue of spatial misalignment. Despite these advances, current fusion strategies—such as simple channel concatenation or stacking, and attention-based weighting mechanisms—often fall short in fully exploiting complementary inter-modal information. Particularly under low-quality feature conditions, these methods may still result in the loss of critical information. To solve the above problems, we combine attention mechanism and Transformer model, and propose an object detection method based on dual-modal fusion network. The dual-branch feature-level fusion method is adopted, and the feature interaction enhancement and fusion module is embedded, so that the model can make full use of dual-modal information.

Our main contributions are as follows:

1) We design a dual-branch backbone to extract features from visible and infrared modalities separately, balancing and mining their unique characteristics to enhance feature richness and specificity.
2) We introduce an interaction enhancement module that dynamically highlights key features by integrating dual-modal information, thereby improving feature saliency and cross-modal complementarity.
3) We propose a fusion network architecture with a Cross Attention mechanism, enabling deep interaction and comprehensive fusion of cross-modal features to significantly boost integration efficiency and detection accuracy.

The remainder of this paper is structured as follows. In Section 2, we describe related works. Section 3 introduces the proposed DEF-Net, including Dual-branch backbone, Cross attention Fusion Network, Object Detection Structure and Loss function. Experiments and discussions are shown in section 4. Subsequently, the conclusion is provided in Section 5.

## 2 Related works

### 2.1 Traditional dual-modal object detection

Traditional dual-modal object detection methods primarily fall into two categories. The first relies on visual feature extraction, utilizing salient characteristics such as edges and color in visible images and brightness in infrared images. Techniques include clustering, threshold-based, and region-based methods. While effective in specific scenarios—such as agricultural detection using color and morphological operations or target recognition through infrared-visible feature fusion—these methods are often sensitive to noise and limited in efficiency and accuracy.

The second category employs handcrafted features with candidate region processing. Candidate regions are generated via sliding windows [13] or selective search [14], followed by feature extraction using algorithms like SIFT [15], HOG [16], ORB [17], or LBP [18], and classification with SVM [19] or Adaboost [20]. Although strategies such as ROI-focused HOG sampling improve efficiency, these methods generally suffer from slow feature extraction, limited generalization, and constrained practical applicability.

### 2.2 Dual-modal object detection based on deep learning

**Fig 1:**
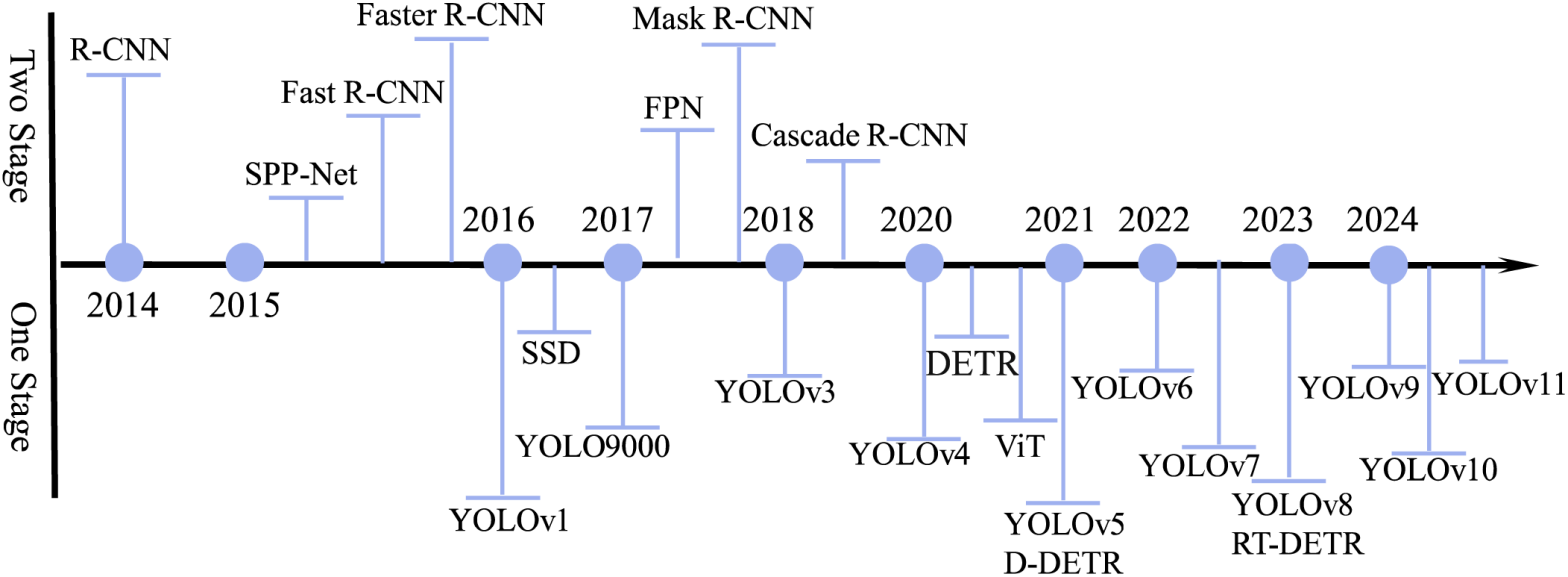
Dual-modal object detection based on deep learning.

Compared to traditional object detection methods, deep learning-based dual-modal object detection utilizes network layers to extract image features, achieving robust feature representations and significantly improving detection performance. The core idea is to adapt and extend existing single-modal detection frameworks through dual-modal fusion and enhancement. Current single-modal detectors can be categorized into two types based on their pipeline: Two-Stage and One-Stage methods, as illustrated in Fig 1.

Two-Stage methods, primarily based on the R-CNN series [21], first generate region proposals using algorithms like selective search, then perform classification and regression on these regions. While achieving high accuracy, these methods suffer from computational redundancy due to overlapping proposals. Fast R-CNN improved efficiency by sharing convolutional features, and Faster R-CNN further replaced selective search with a Region Proposal Network (RPN), reducing redundancy and increasing speed [22]. Despite their accuracy, Two-Stage methods are often outperformed in practical applications by One-Stage approaches due to their superior inference speed and competitive performance.

One-Stage methods, such as SSD, YOLO, and DETR, perform end-to-end detection by directly predicting object categories and bounding boxes from the input image, eliminating the need for region proposal generation [23–30]. SSD uses multi-scale feature maps for detection but struggles with small objects due to insufficient shallow feature utilization. The YOLO series [31], built on Darknet, divides the image into grids and predicts objects within each cell. YOLOv3 introduced multi-scale detection, YOLOv5 improved efficiency and ease of deployment [32], and later versions like YOLOv8 and YOLOv10incorporated advanced attention mechanisms and end-to-end strategies [33–34]. YOLOv11 further reduced parameters while maintaining performance. Despite progress, detecting small and dense objects remains challenging.

Transformers, initially designed for NLP, have also been adapted for vision tasks. DETR [35] pioneered the use of Transformer encoder-decoder architecture for object detection, using object queries to directly output predictions without non-maximum suppression. While accurate, it suffers from high computational cost. RT-DETR enhanced real-time performance with a hybrid encoder and IoU-aware query selection, offering a better speed-accuracy balance for practical use.

The existing dual-modal object detection algorithms based on visible and infrared images have not fully exploited the complementarity and rich context information between different modalities. Therefore, there is still a broad research space to improve the performance of bimodal object detection, especially in complex scenes. This study will explore a more effective mechanism for deep interaction between features of different modalities, so as to further improve the accuracy and generalization ability of bimodal object detection.

## 3 Method

**Fig 2:**
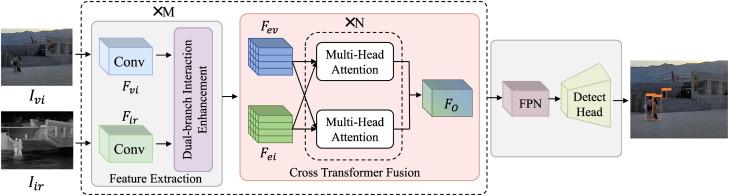
Overall framework of the proposed DEF-Net.

The specific flow chart of the proposed dual-modal fusion network (DEF-Net) is show in Fig 2, which mainly includes three parts, Dual-branch backbone, Cross fusion network and Object Detection Structure.

1) The Dual Branch Backbone: It is composed of the Convolutional Feature Extraction and the Dual-branch Interaction Enhancement. The visible image $I_{vi}$ and infrared image $I_{ir}$ obtain features $F_{vi}$ and $F_{ir}$ through the Feature Encoder respectively.
2) Cross-Transformer Fusion: $F_{ev}$ and $F_{ei}$ are obtained more effective feature representation through the cross-attention network layer to obtain fusion features $F_{O}$, which realizes the complementarity of dual-modal feature information.
3) Object Detection Structure: The output features of the first two parts are used as the input of the Feature Pyramid Networks, and sent to the detection head to obtain the classification and localization of the target.

### 3.1 Dual-branch backbone

Dual-branch backbone network is composed of Convolutional Feature Extraction and the Dual-branch Interaction Enhancement.

#### 3.1.1 Convolutional feature extraction.

**Fig 3:**
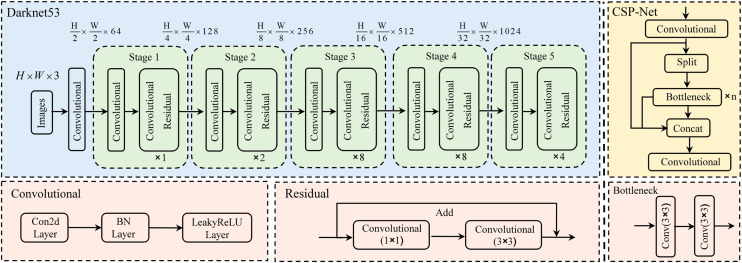
The backbone network architecture of Darknet53.

The dual-branch backbone network aims to extract and represent the features of infrared and visible data, and lay the foundation for feature fusion. Darknet53, as a network backbone architecture based on residual learning, becomes a suitable framework for bimodal object detection tasks due to its unique structural design and advantages, as shown in Fig 3.

Firstly, the network uses a 3 × 3 convolution layer with a step size of 2 for feature extraction, and then concatenates 1 × 1 and 3 × 3 convolution operations, and forms a residual connection with the convolution layer feature map. Through this deep superposition of structural units including 1 × 1 convolutions, 3 × 3 convolutions and residual edges, the network is deepened and the expression ability of the network is significantly enhanced. In addition, each convolutional module adopts the unique DarknetConv2D structure, including l2 regularization, Batch Normalization, and ReLU activation function. This design not only optimizes the training process of the network, improves the performance of the model by increasing the depth of the network, but also effectively alleviates the problem of gradient disappearance in deep neural networks by means of skip connections. However, simply extending the single-branch model to a two-branch structure leads to a significant increase in computational resources. In response to this challenge, we introduce Cross Stage Partial Network (CSPNet) to optimize the network structure and calculation process, which can significantly improve the feature learning ability of lightweight models. The core idea is that the gradient information can be propagated through different network paths through the segmentation mechanism of the gradient flow. By switching the series calculation and transition steps, the propagated gradient information can obtain a larger correlation difference, so as to significantly reduce the computational complexity and improve the inference efficiency and detection accuracy.

**Fig 4:**
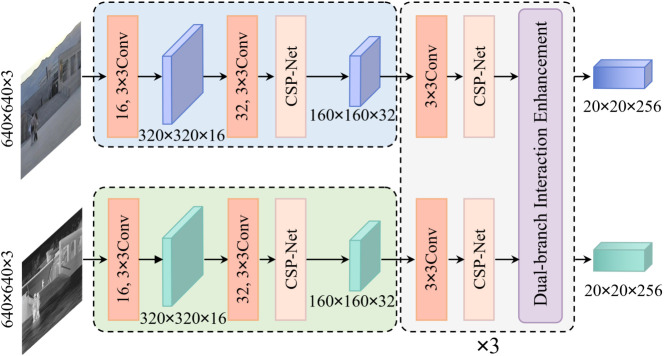
Dual-branch backbone structure.

Referring to the CSPDarknet53 backbone network [36], the double-branch backbone network is designed as shown in Fig 4, including CONV module, CSP structure and DBE module. CONV module was used as the basic module of feature extraction, including convolutional layer, batch normalization layer and SILU activation function. The CSP structure introduces residual features to propagate gradient information and enhance the learning ability of the convolutional network. The DBE module combines the dual-branch features to obtain the dual-modal feature map of the enhanced representation. In order to balance the dual-modal information and limit the complexity of the network, both branches use 640 × 640 pixels, 3 channels of input, and finally are downsampled to 20 × 20 × 256 feature maps. The design aims to fully extract and enhance the bimodal information, and provide a favorable feature representation basis for subsequent feature fusion.

#### 3.1.2 Dual-branch interaction enhancement.

**Fig 5:**
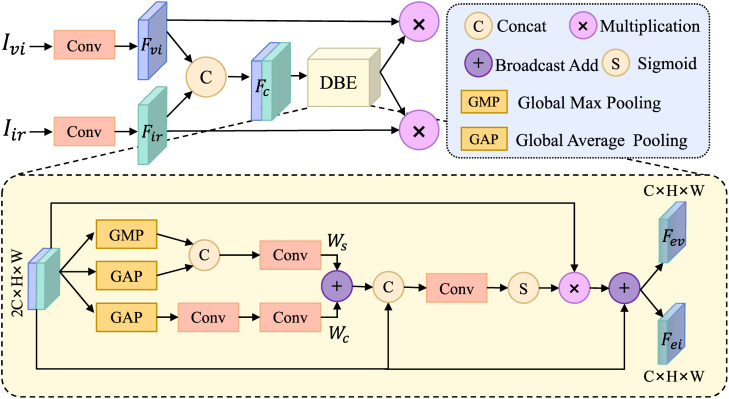
Feature interaction and enhancement structure.

At present, most of the methods to improve the performance of feature extraction are to fuse features by using various types of attention, including channel attention and convolutional attention. However, the intra-modal attention can only solve the uneven distribution of single-modal features, but ignores the uneven distribution at the bi-modal feature level. Although the channel attention inside the feature attention module integrates the differences between channels, it does not consider the context information between infrared and visible light, which leads to an unbalanced distribution of infrared and visible light information in each feature channel in the spatial dimension. In addition, the attention weights of space and channel in feature attention usually lack information interaction. Therefore, it is necessary to combine the feature channels of infrared and visible to construct a spatially important feature map, and achieve the enhancement of bimodal information through feature interaction. In order to solve the above problems. Dual-branch Interaction Enhancement (DBE) is shown in Fig 5.

The proposed DBE addresses these limitations by introducing a parallel dual-attention mechanism that enables simultaneous spatial and channel-wise enhancement of bimodal features. Through broadcast addition, the module facilitates early interaction between spatial and channel attentions, generating adaptive fusion weights. These weights are further refined with original features to produce channel-specific spatial interaction maps, which dynamically highlight important regions across modalities while preserving original information via residual connections. This design ensures effective bimodal feature interaction and enhancement at the attention level, providing a more discriminative feature representation for subsequent fusion.

After a series of convolution modules, the visible and infrared features of $I_{vi}$, $I_{ir}$ are obtained as $F_{vi},F_{ir}\in {\text{R}}^{C\times H\times W}$. In order to use the bimodal information, $F_{vi},F_{ir}$ after Concat $F_{c}\in {\text{R}}^{2C\times H\times W}$ is used as the input of DBE to calculate the spatial attention and channel attention. See Eq (1) and Eq (2) to obtain the spatial attention weights $W_{s}\in {\text{R}}^{H\times W}$ and $W_{c}\in {\text{R}}^{2C\times 1\times 1}$.


$$W_{s}={\text{Conv}}_{7\times 7}\left(\text{Concat}\left(\text{MaxPool}\left(F_{c}\right),\text{AvgPool}\left(F_{c}\right)\right)\right)$$ (1)

$$W_{c}={\text{Conv}}_{1\times 1}\left(\text{Max}\left(0,{\text{Conv}}_{1\times 1}\left(\text{AvgPool}\left(F_{c}\right)\right)\right)\right)$$ (2)

In Eq (1), AvgPool represents the global average pooling across channel dimensions, and MaxPool represents the global maximum pooling operation across channel dimensions. In Equation (2), AvgPool represents the global average pooling across spatial dimensions, and the two convolutions respectively reduce the dimension and increase the dimension. The first convolution reduces the dimension 2C to 2C/r, *r* is the scaling factor, and the second convolution expands it back to 2C. In order to limit the complexity of the model, when *r* is set to 8, the calculation amount is maintained and the effect is considerable. The activation function is introduced in the two convolutions to improve the feature representation and help the gradient flow. In order to make the two kinds of attention interact, the Broadcast mechanism is used to add $W_{c}$ and $W_{s}$ to obtain the initial attention weight $W_{cs}$, and the formula is as follows.


$$W_{cs}=W_{c}+W_{s}$$ (3)

The initial attention weight $W_{cs}$ is refined according to each channel of the input bimodal feature map to focus on a unique part of the features in each channel to generate the final SIM. The feature layer after concatenating the input dual-modal feature map $F_{c}$ and the attention weight $W_{cs}$ is used as the input of the convolution layer, and the feature weight value is normalized by the sigmoid function to obtain *W*, which is calculated as shown in Equation (4). *W* combines the dual-modal feature information and assigns a unique SIM to each channel. To guide the model to focus on important regions in the infrared and visible light channels.


$$W=\sigma \left({\text{Conv}}_{7\times 7}\left(\text{Concat}\left(\left[F_{c},W_{cs}\right]\right)\right)\right)$$ (4)

The weighted feature information is calculated by combining W and input features, and the residual connection is introduced to add input features, as shown in Eq (5), so as to enhance the gradient transfer and prevent the loss of feature information caused by too low weight assignment.


$$F_{f}=F_{c}\cdot W+F_{c}$$ (5)

Finally, the features are assigned according to the channel dimension to obtain the interactive enhanced bimodal features, as shown in Eq (6).


$$\left\{ \begin{array}{c} @l@F_{ev}=\text{Concat}\left(\left[F_{fi}\right]\right),i=1,2,...,C\;\\ F_{ei}=\text{Concat}\left(\left[F_{fj}\right]\right),j=C+1,...,2C \end{array}\right.$$ (6)

After dimension assignment, the enhanced features $F_{ev}$ and $F_{ei}$ are obtained and used as the input of the subsequent fusion module.

### 3.2 Cross attention fusion network

**Fig 6:**
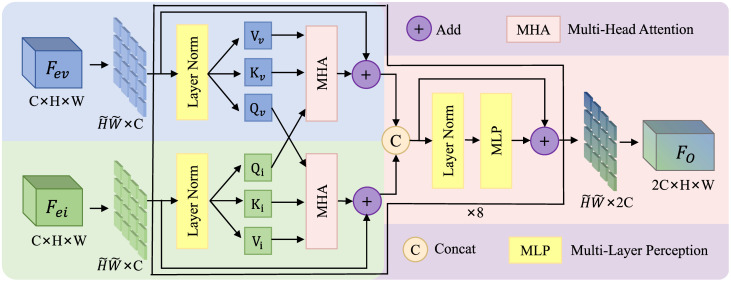
Cross attention fusion network structure.

The interaction enhancement module based on spatial and channel attention mechanisms enables the model to focus on important features. However, only assigning weights to bimodal feature channels cannot effectively exploit the complementarity between the two modalities. In order to fully mine and utilize the complementary information of infrared and visible light features, a Cross-Attention fusion network is constructed by using the advantage of Transformers in modeling long-range sequence relationships and the idea of cross-attention Transformers (CAT). The structure is shown in Fig 6.

The CAFN employs a bidirectional query-retrieval mechanism to achieve deep cross-modal complementarity: infrared features serve as queries to retrieve relevant visible information, while visible features query complementary infrared content. This dynamic retrieval process is further enhanced through multi-head attention, which captures diverse cross-modal correlations across different representation subspaces. The resulting fused features exhibit both semantic consistency and detail completeness, effectively integrating complementary information from both modalities. By structuring fusion as an interactive retrieval process, CAFN enables more robust and discriminative feature representations for downstream detection tasks.

In the feature maps of infrared and visible light, useful information is mutually retrieved and extracted, while interfering information remains inactive. This essentially constitutes a bidirectional complementary information extraction process—mutual information retrieval from visible to infrared and from infrared to visible light—to fuse dual-modal features. Specifically, given the interactively enhanced visible and infrared features $F_{ev},F_{ei}\in {\text{R}}^{C\times H\times W}$, in order to utilize Transformer-based attention, the inputs are downsampled via average pooling and undergo dimension transformation to convert them into sequences $I_{ev},I_{ei}\in {\text{R}}^{\tilde{H}\times \tilde{W}\times C}$, To constrain model complexity, we set $\tilde{H}=\tilde{W}=8$. For the input sequences, they are projected into three weight matrices to compute a set of Query, Key, and Value vectors, as shown in Eq (7) to (9).


$$Q_{i}=I_{ei}W^{Qi}$$ (7)

$$K_{v}=I_{ev}W^{Kv}$$ (8)

$$V_{v}=I_{ev}W^{Vv}$$ (9)

In Eq (7) to (9), ${\text{W}}^{Qi}\in {\text{R}}^{C\times D_{Q}},{\text{W}}^{Kv}\in {\text{R}}^{C\times D_{K}},{\text{W}}^{Vv}\in {\text{R}}^{C\times D_{V}}$ are learnable weight parameters. In this model, the number of channels for both modalities is C, and the parameters are set as$D_{Q}=D_{K}=D_{V}=C$. The infrared feature information is used to retrieve visible feature information to compute channel correlations. The cross-attention weights are calculated through scaled dot-product operations. In this process, the visible feature information provides the Key and Value, as shown in Eq (10). The output $C_{V}$ represents the visible attention weights fused with infrared features.


$$C_{V}=\text{CrossAttention}\left(Q_{i},K_{v},V_{v}\right)=\text{softmax}\left(\frac{Q_{i}{K_{v}}^{T}}{\sqrt{D_{K}}}\right)V_{v}$$ (10)

**Fig 7:**
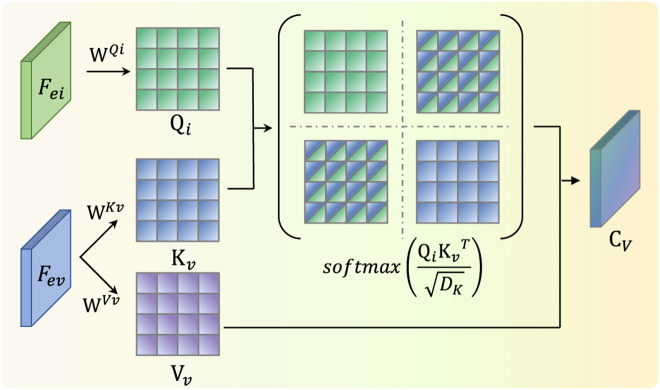
Illustration of Cross attention weight.

The cross-attention mechanism enables adaptive fusion of visible and infrared image features across different representation spaces. By automatically performing both intra-modal and inter-modal information fusion, this mechanism computes a correlation matrix between features to capture potential interactions between the visible and infrared modalities. These relationships are quantified into corresponding attention weights $C_{V}$, thereby achieving effective and complementary feature fusion. A schematic diagram of this computation is shown in Fig 7.

In order to deal with the representation of different complex relations from different locations, the multi-head attention mechanism is adopted, as shown in Eq (11).


$$\overset{M}{\underset{V}{C}}=\text{MHA}\left(Q_{i},K_{v},V_{v}\right)=\text{Concat}\left(C_{V1},\ldots ,C_{Vh}\right){\text{W}}^{O}$$ (11)

Where h denotes the number of heads, which is set to 4 in our method. ${\text{W}}^{O}\in {\text{R}}^{h\cdot C\times C}$ represents the parameter matrix after concatenating $\left(C_{\text{1}},\ldots ,C_{h}\right)$, and is used to generate the fused infrared-visible feature attention weights $\overset{M}{\underset{V}{C}}$. Similarly, the corresponding weights $\overset{M}{\underset{I}{C}}$ can be obtained for the infrared features. The two sets of weights are applied to the initial branch features, respectively, and then concatenated to form $\overset{M}{\underset{F}{S}}$. The fused feature $F_{O}$ is output through a subsequent Transformer connection layer, as shown in Eq (12):


$$F_{O}\text{\textasciitilde{}=\textasciitilde{}MLP}\left(\overset{M}{\underset{F}{S}}\right)+\overset{M}{\underset{F}{S}}{\text{= FC}}_{\text{2}}\left(\text{RELU}\left({\text{FC}}_{\text{1}}\left(\overset{M}{\underset{F}{S}}\right)\right)\right)+\overset{M}{\underset{F}{S}}$$ (12)

The Transformer connection layer consists of a two-layer neural network, which sequentially passes through a linear layer FC₁, ReLU non-linear activation, and another linear transformation FC₂. As illustrated in Fig 5, the fusion module contains 8 cross-attention blocks to achieve inter-modal fusion. The final output corresponds to the input feature dimensions, and the output $F_{O}$ is upsampled to the original resolution via bilinear interpolation. By leveraging the cross-attention mechanism of the Transformer, the model can naturally perform inter-modal fusion and effectively capture interactive relationships between visible and infrared features, thereby enhancing the performance of dual-modal object detection.

### 3.3 Object detection structure and loss function

#### 3.3.1 Object detection structure.

The backbone of the network consists of two parts: the dual-branch backbone network and the fusion module described above. The dual-branch backbone network progressively enhances dual-modal features through multiple feature interaction blocks, while the feature fusion module enables cross-modal fusion at various scales. In the paper, the outputs of the backbone are as follows:

- A 32 × downsampled dual-modal feature map obtained after 3 layers of DBE feature enhancement and fusion module processing,
- A 16 × downsampled dual-modal feature map formed by concatenating features after 2 layers of DBE enhancement,
- An 8 × downsampled dual-modal feature map formed by concatenating features after 1 layer of DBE enhancement.

These three multi-scale feature maps are fed into a Feature Pyramid Network (FPN) to improve object detection performance by integrating both high-level and low-level features. Finally, the three levels of features are processed by the YOLOv8-Head to output the final bounding box regressions and classification results.

#### 3.3.2 Loss function.

The loss function of the proposed method consists of two parts: the bounding box loss for object detection and the improved cross-entropy loss for classification.

The bounding box loss uses Complete-IoU Loss (CloU), which comprehensively considers three key metrics: overlap area, center point distance, and aspect ratio. By constructing the loss function to bring predicted boxes closer to ground truth boxes, CloU enables more thorough optimization. The CloU calculation is given by Eqs (13) – (15):


$$\text{CIoU}=\text{IoU}-\left(\frac{\rho^{\text{2}}\left(b,b^{gt}\right)}{c^{\text{2}}}\right)+\alpha \nu$$ (13)

$$\nu =\frac{\text{4}}{\pi^{\text{2}}}-(\text{arctan}\frac{w^{gt}}{h^{gt}}-\text{arctan}\frac{w}{h}{)}^{\text{2}}$$ (14)

$$\alpha =\frac{\nu }{\left(1-\text{IoU}\right)+\nu }$$ (15)

Where, IoU denotes the intersection over union between the predicted and ground truth boxes, *ρ* represents the Euclidean distance between their center points, *c* is the diagonal length of the smallest enclosing box covering both boxes, and *α* is a balancing coefficient. The terms *b* and $b^{gt}$ refer to the center points of the predicted box and ground truth box, respectively. The variables *w*, *h*, $w^{gt}$, and $h^{gt}$ denote the width and height of the predicted box and the ground truth box, while *v* measures the consistency of aspect ratio between the two boxes.

The classification loss employs Varifocal Loss (VFL), which adopts an asymmetric weighting scheme for training samples. It reduces the weight of negative samples while increasing the weight of high-quality positive samples, thereby focusing training on high-quality positives. The loss is computed as shown in Eq (16).


$$\begin{array}{c} VFL\left(p,q\right)=\left\{ \begin{array}{cc} @cc@-q\left(q\text{log}\left(p\right)+\left(1-q\right)\text{log}\left(1-p\right)\right) & q>0\\ -\alpha p^{\gamma }\text{log}\left(1-p\right) & q=0 \end{array}\right. \end{array}$$ (16)

Here, *p* is the predicted classification score, and *q* is the IoU between the predicted box and the ground truth box. When *q* > 0, the sample is considered positive. If the two boxes do not overlap, *q* = 0, and the sample is treated as negative. The terms *α* and *γ* are hyperparameters. This loss function places greater emphasis on hard-to-classify positive samples, thereby improving overall object detection performance.

## 4 Experiment and analysis

### 4.1 Experimental environment and training strategy

All models were trained on a system running Windows 11 23H2. The hardware platform consisted of an Intel Core i7-13650 CPU and an NVIDIA GeForce GTX 4060 GPU, with CUDA version 11.6. All experiments were implemented using Python 3.8 and the PyTorch 1.13.1 framework. No pre-trained weights were loaded during training to ensure a fair comparison across different architectures.

The detailed hyperparameter settings used in our experiments are summarized in Table 1. We adopted Stochastic Gradient Descent (SGD) with momentum as the optimizer, with an initial learning rate of 0.01 and a weight decay coefficient of 5 × 10 ⁻ ⁴. The learning rate was reduced by a factor of 0.1 at epochs 50, 100, and 150. The batch size was set to 8 due to GPU memory constraints. During training, a visualization tool was employed to monitor the loss function, which stabilized after approximately 200 epochs, indicating model convergence.

**Table 1:** Training Hyperparameter Configuration.

Hyperparameter	Setting	Description
framework	PyTorch 1.13.1	with CUDA 11.6 support
Pre-trained Weights	None	trained from scratch
Weight Initialization	Kaiming Normal	for ReLU-based layers
Optimizer	SGD with Momentum	momentum = 0.9
Weight Decay	5 × 10 ⁻ ⁴	Regularization coefficient
Initial Learning Rate	0.01	base learning rate
LR schedule	Step decay	×0.1 at epochs 50, 100, 150
Batch Size	8	limited by GPU memory
Total Epochs	200	training converges around this point
Data Augmentation	Random Horizontal Flip	probability = 0.5
Test Batch Size	16	No gradient computation

### 4.2 Datasets and evaluation indicators

**Fig 8:**
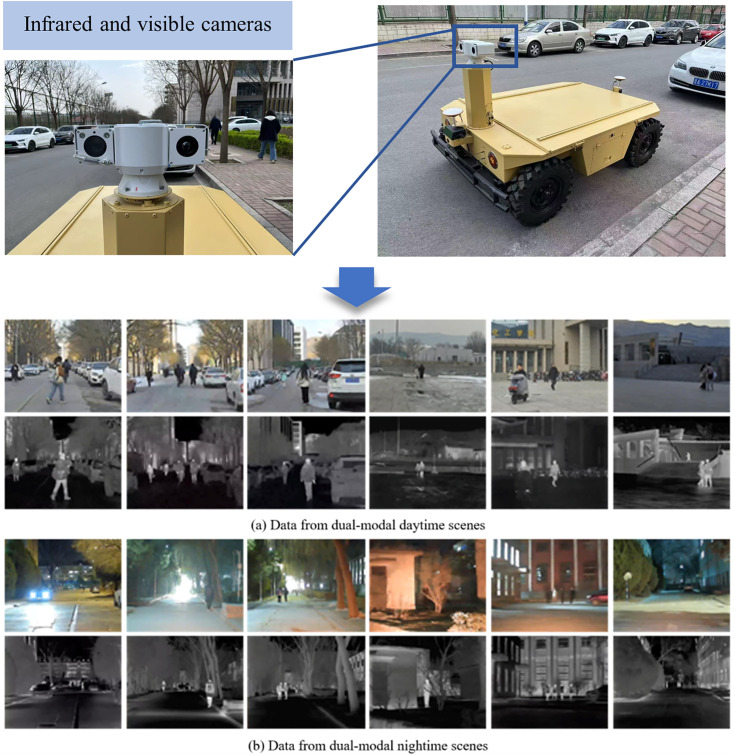
SYUGV Datasets.

To validate the effectiveness of the proposed method in complex dual-modal detection scenarios, we constructed the SYUGV dataset using our unmanned ground vehicle platform SYUGV-01, equipped with synchronized infrared and visible cameras (30 fps, 720 × 576 resolution). The dataset contains 6,272 image pairs (3,232 daytime, 3,040 nighttime) covering challenging conditions such as nighttime, overexposure, motion blur, and occlusion (as shown in Fig 8).

We applied a coarse feature-based registration method with an alignment accuracy within ~10 pixels, which provides region-level semantic consistency suitable for object detection, consistent with common practice in multimodal detection benchmarks. All image pairs share COCO-format annotations across two categories (pedestrians, vehicles) and are split into 5,146 training and 1,126 test pairs with no overlap. The dataset will be publicly released upon acceptance to support reproducibility. Additionally, we evaluate our method on the public LLVIP dataset [37] for generalization assessment.

The evaluation metrics used in the experiments include Precision (P), Recall (R), mean Average Precision (mAP), and Frames Per Second (FPS). Additionally, Floating Point Operations (FLOPs) and the number of model parameters (Params) were used to assess algorithm complexity.

### 4.3 Comparative experiment and analysis

In order to verify the effectiveness of the algorithm in this chapter, it is applied to the SYUGV and LLVIP datasets with the recent bimodal object detection algorithms, such as MAF-YOLO [10], SLBIF-Net [11], and ICAFusion [12], and the indicators are used for evaluation and comparison.

#### 4.3.1 Results and analysis of SYUGV dataset.

To validate the performance of the proposed method under realistic operational scenarios, we conducted comprehensive experiments on our self-built SYUGV dataset. This dataset emulates challenging conditions encountered by unmanned platforms in the field (e.g., low-light, occlusion, and motion blur), providing an in-depth evaluation of accuracy, robustness, and real-time capability.

1) Quantitative Performance

**Fig 9:**
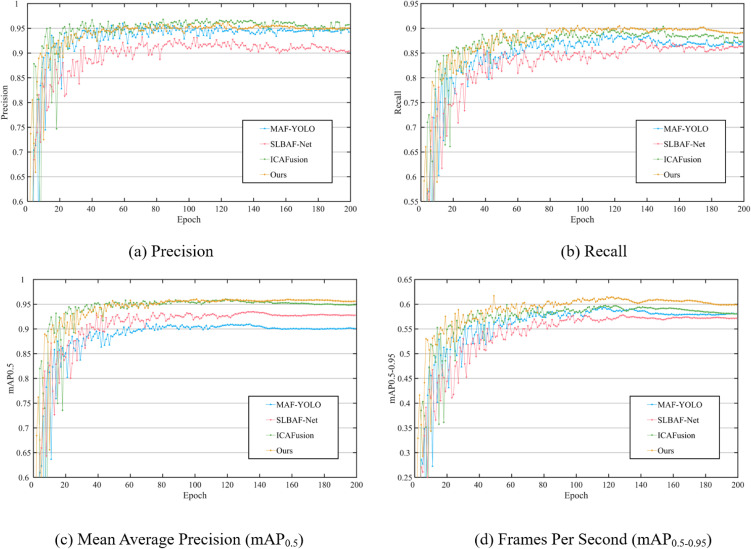
Comparison of model training on the SYUGV dataset.

As shown in Fig 9 and Table 2, the proposed DEF-Net achieves state-of-the-art results on the SYUGV test set. The model attains the highest mAP@0.5 of 95.64%, outperforming MAF-YOLO (92.76%) and SLBAF-Net (93.13%) by 2.88% and 2.51%, respectively. This improvement can be attributed to the synergistic enhancement of salient features via the Dual-branch Interaction Enhancement (DBE) module and the deep integration of complementary information through the Cross-Attention Fusion Network (CAT).

**Table 2:** Comparative experimental results of different models.

Method	P/%	R/%	mAP_0.5_/%	mAP_0.5–0.95_/%	Params/M	FPS
MAF-YOLO [10]	93.52	85.38	92.76	64.42	6.1	63
SLBAF-Net [11]	88.47	87.33	93.13	61.76	4.1	71
ICAFusion [12]	94.03	90.61	95.04	64.89	23.2	26
Ours	95.26	90.48	95.64	69.63	12.3	117

In particular, the model achieves a recall (R) of 90.48%, the highest among all compared methods, indicating a lower miss-detection rate. This demonstrates the effectiveness of the CAT module in retrieving missing features from the complementary modality. Under the stricter mAP@0.5:0.95 metric, DEF-Net also leads with 69.63%, reflecting more accurate bounding-box localization. Importantly, while maintaining high accuracy, DEF-Net reaches an inference speed of 117 FPS, significantly exceeding other methods and satisfying real-time detection requirements.

2) Qualitative Analysis

**Fig 10:**
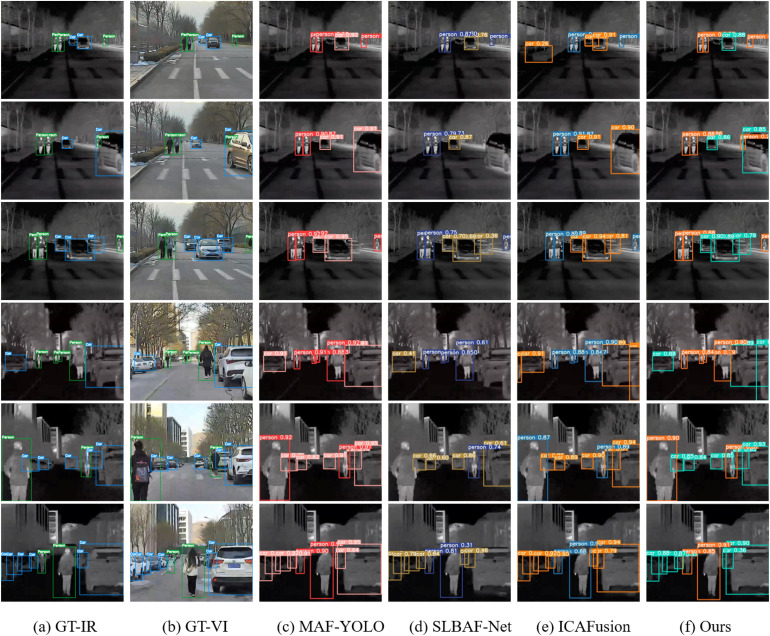
Comparison of detection effects of different models on the SYUGV dataset.

Fig 10 displays detection results on six challenging scenes from the SYUGV dataset, visualized on infrared images for clearer target contrast. Inference thresholds were fixed at 0.25 (confidence) and 0.7 (IoU) for all methods.

The qualitative results demonstrate the robustness of our DEF-Net. In low-light or nighttime scenes (e.g., Scene 1 and Scene 4), DEF-Net successfully detects several pedestrians that are missed by other methods (highlighted with yellow circles), which visually substantiates the effectiveness of the Cross-Attention Fusion Network (CAT) in leveraging thermal information to reinforce features from the visible spectrum. In scenarios involving occlusion or target interaction (e.g., Scene 2 and Scene 3), DEF-Net produces more complete bounding boxes around partially obscured targets while generating fewer false positives. This can be attributed to the Dual-branch Interaction Enhancement (DBE) module, which dynamically emphasizes spatially and semantically informative regions within the feature maps.

**Fig 11:**
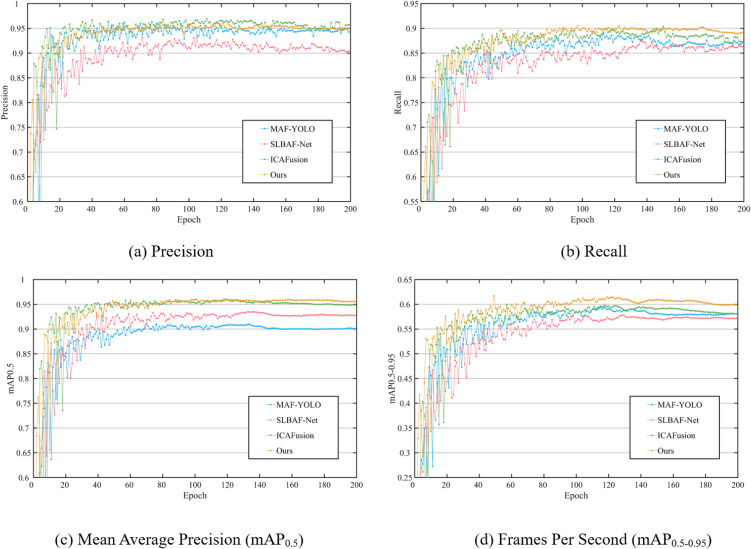
Comparison of model training on the LLVIP dataset.

These visual results align with the quantitative metrics in Table 2, confirming DEF-Net’s robustness and practical suitability for real-time detection in complex environments.

#### 4.3.2 Results and analysis of LLVIP dataset.

To assess the generalization capability and benchmark performance of the proposed method, we further evaluated DEF-Net on the public LLVIP dataset.

1) Quantitative Performance

The quantitative results in Fig 11 and Table 3 demonstrate that DEF-Net remains highly competitive on this independent benchmark. Although its precision (95.39%) is marginally lower than that of MAF-YOLO (96.09%) and ICAFusion (96.53%), our method achieves the highest recall (89.51%), indicating a stronger ability to reduce missed detections through enhanced cross-modal complementarity. More importantly, DEF-Net obtains the best overall accuracy, with mAP@0.5 of 95.70% (exceeding ICAFusion by 0.87%) and mAP@0.5:0.95 of 61.67% (surpassing ICAFusion by 1.80%). These results confirm that DEF-Net effectively balances precision and recall, leading to superior comprehensive detection performance. Notably, DEF-Net maintains an excellent balance between model complexity and accuracy. Compared with the high-accuracy ICAFusion, our method uses approximately 47% fewer parameters (12.3 M vs. 23.2 M) and runs 4.7 times faster (113 FPS vs. 24 FPS), making it markedly more suitable for real-time deployment in practical systems.

**Table 3:** Comparative experimental results of different models.

Method	P/%	R/%	mAP_0.5_/%	mAP_0.5–0.95_/%	Params/M	FPS
MAF-YOLO [10]	96.09	86.74	90.34	57.24	6.1	61
SLBAF-Net [11]	92.38	86.31	92.94	57.16	4.1	70
ICAFusion [12]	96.53	88.52	94.83	59.87	23.2	24
Ours	95.39	89.51	95.70	61.67	12.3	113

It is worth emphasizing that the performance trends observed on SYUGV—specifically, superior recall and leading mAP—are consistently reproduced on LLVIP. This cross‑dataset coherence reinforces the generalizability and reliability of the proposed architecture.

2) Qualitative Validation

**Fig 12:**
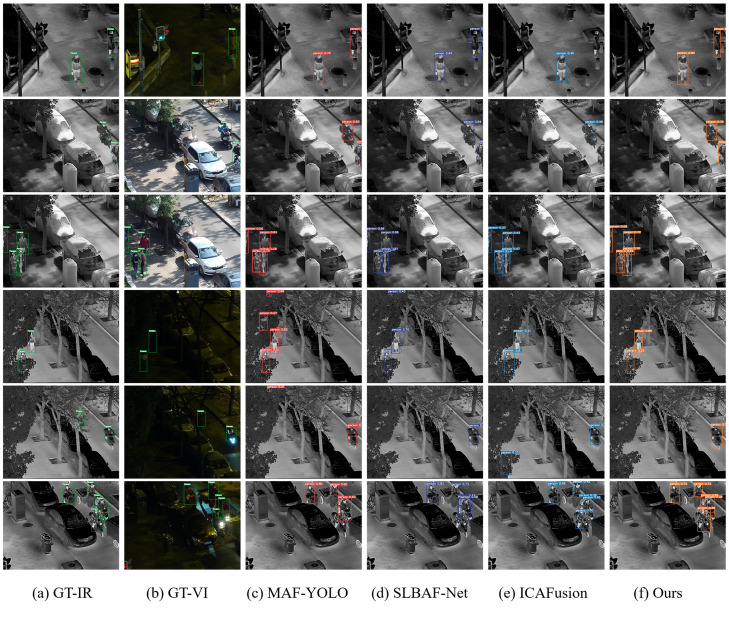
Comparison of detection effects of different models on the LLVIP dataset.

For a visual comparison, Fig 12 presents detection results on six representative scenes from LLVIP. To ensure clarity, all results are overlaid on the infrared modality, where target information is typically more distinct. During inference, the confidence threshold and the Intersection over Union (IoU) threshold were uniformly set to 0.25 and 0.7, respectively. The visualizations show that DEF-Net produces more complete and accurate bounding boxes across diverse scenarios, further corroborating the quantitative advantages reported in Table 3.

From the above experimental results, it can be seen that our method shows good performance on the bimodal object detection task. Compared with other methods, our method maintains a relatively stable detection effect in different scenes and conditions, especially in the case of occlusion (scene 4 and scene 6) and illumination change (scene 2 and scene 3), it still maintains a good detection effect.

### 4.4 Ablation study

#### 4.4.1 Number of feature channels in backbone network.

In order to limit the complexity of the model while maintaining accuracy, Yolov8s is selected as the single-modal benchmark detection network in this study. The dual-modal benchmark network modifies the Backbone part of the Darknet to the above dual-branch backbone network structure, reduces the number of feature channels, does not use the DBE module, and Concat the infrared features and visible features through the convolutional layer to obtain the fusion features and send them to the detection head. Thus, the Dual-CSPDarkNet (hereinafter referred to as Dual-CDNet) is constructed, which will be used as the dual-branch benchmark model for subsequent experiments to verify the effectiveness of the method proposed in this paper.

**Fig 13:**
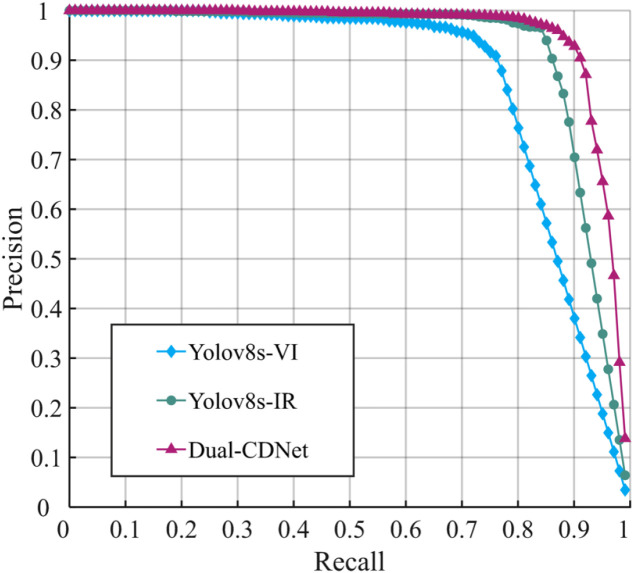
P-R curves of different modal inputs.

Through the experimental comparison on the SYUGV dataset, as shown in Table 4, it is verified that the dual-branch backbone network improves the detection performance. Specifically, the mAP0.5 of YOLOv8s model is 86.74% when only visible light image (VI) is used as input, and the mAP0.5 is 91.82% when only infrared image (IR) is used as input. The average detection accuracy of dual-modal images based on the dual-branch backbone network reaches 94.06%, which is 7.32%and 2.24%higher than that of the visible and infrared image input alone. At the same time, by reducing the number of backbone network feature channels, Flops and Params are respectively reduced by 5.8G and 2.3M compared with the single-branch benchmark model on the Dual-CDNet model. In terms of detection speed, the proposed dual-branch model structure maintains the detection speed of the single-branch structure. Fig 13 plots the Precision-Recall (P-R) curves for the different models. It can be intuitively seen that after using the dual-branch structure to extract the infrared and visible light features respectively, the overall detection accuracy is significantly improved compared with the single mode.

**Table 4:** Model detection performance of different modal inputs.

Method	Input	P/%	R/%	mAP_0.5_/%	mAP_0.5–0.95_/%	Flops/G	Params/M	FPS
Yolov8s	VI	89.13	78.17	86.74	54.14	28.6	11.1	150
Yolov8s	IR	91.90	86.33	91.82	65.34	28.6	11.1	150
Dual-CDNet	VI + IR	93.17	88.04	94.06	68.94	22.8	8.8	163

#### 4.4.2 The detection performance of dual branch model.

**Fig 14:**
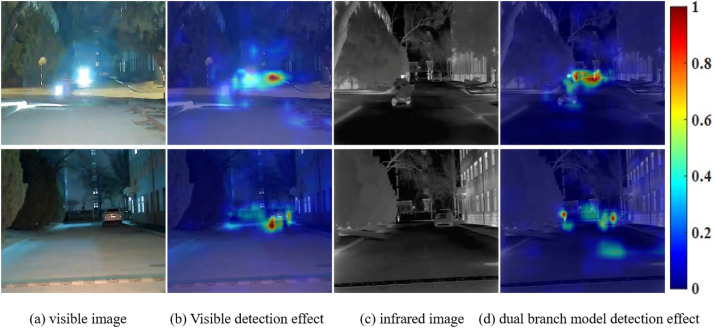
Grad-CAM heatmap of dual branch model.

The Grad-CAM method is used to identify and highlight the key regions in the input image for category prediction of the network model. Select all the target categories and use the feature map to calculate the weight features for different layers to generate a heat map. In the resulting heatmap, the red regions indicate the regions that the model focuses on when making a prediction, that is, the regions that have the most influence on the prediction result, the yellow regions have the second most attention, and the blue regions have the least influence on the prediction result. In this chapter, night scene data is taken as an example, and different network layers are selected by Grad-CAM method to realize feature attention visualization, as shown in Fig 14.

As can be seen from Fig 14, when dealing with nighttime images, especially in the case of local overexposure or insufficient light, as shown in Fig 14 (a), the visible light image is difficult to show all the target information, and the visible single-branch model only focuses on some weak features, as shown in Fig 14 (b). After the infrared branch is introduced, the model significantly focuses on the brightness characteristics of thermal targets that are difficult to detect in visible images but prominent in infrared images, as shown in Fig 14 (d). The dual-branch backbone network was used to process the images of two modes respectively, and the specific characteristics of infrared and visible light were used, which verified the effectiveness of the dual-branch structure in processing dual-modal information.

Due to the modal heterogeneity of infrared and visible, independent networks are required to extract the features of both respectively, thus constructing the above two-branch backbone and demonstrating its effectiveness. In addition, since the number of parameters and computational complexity of different backbone networks are different, experiments are needed to evaluate the performance of the backbone network. This paper selects several current mainstream backbone models to construct corresponding dual-branch structures. Six dual-branch model architectures—Dual-EfficientNet, Dual-DenseNet, Dual-MobileNet, Dual-SwinT, Dual-RTDETR, and Dual-CDNet—are built based on EfficientNet-B0 [38], DenseNet-121 [39], MobileNet-v3 [40], SwinTransformer-T [41], RT-DETR-R18 [42], and CSPDarkNet, respectively. To objectively evaluate the performance of each dual-branch model, comparative experiments were conducted on the SYUGV dataset using both infrared and visible modalities as input. Detailed metric results are shown in Table 5.

**Table 5:** Model detection performance of different backbone networks.

Method	Input	P/%	R/%	mAP_0.5_/%	mAP_0.5–0.95_/%	Params/M	Flops/G
Dual-DenseNet	VI + IR	93.51	89.49	94.40	68.85	16.6	102.2
Dual-MobileNet	VI + IR	90.62	86.65	93.44	67.41	4.2	18.2
Dual-EfficientNet	VI + IR	93.11	88.06	93.54	68.40	14.6	20.9
Dual-SwinT	VI + IR	93.95	86.93	94.42	66.10	28.6	43.0
Dual-RTDETR	VI + IR	90.32	87.05	92.51	63.21	18.4	34.2
Dual-CDNet	VI + IR	93.17	88.04	94.06	68.94	8.8	22.8

It can be found that the Dual-CDNet model built on CSPDarkNet shows significant advantages. The model achieves a better balance between computing resource consumption and detection accuracy. Compared with models such as Dual-SwinT and Dual-RTDETR with Transformer backbone network, Models based on CNN architectures, including Dual-DenseNet, Dual-MobileNet, Dual-EfficientNet, and Dual-CDNet, among others, have clear advantages in terms of computational overhead while maintaining comparable levels of performance. This is because the data size limit of the current object detection task fails to give full play to the advantages of the Transformer architecture. The Dual-CDNet model has a more flexible feature hierarchy than other models based on CNN architecture, which makes it better adapt to the subsequent interactive fusion module, and provides an ideal infrastructure support for the fusion of dual-modal features. Therefore, this paper chooses the Dual-CDNet model as the basic framework model to provide architectural support for the subsequent fusion research of dual-modal features.

#### 4.4.3 Contribution analysis of the proposed modules.

To systematically evaluate the individual and synergistic contributions of the proposed Dual-branch Interaction Enhancement (DBE) module and the Cross-attention Fusion (CAT) module, a comprehensive ablation study was conducted on both the SYUGV and LLVIP datasets. The baseline model (Baseline) employs a dual-branch backbone with simple feature concatenation.

1) Quantitative Analysis

The detailed results are summarized in Table 6 (for SYUGV) and Table 7 (for LLVIP). Several key observations can be made:

**Table 6:** Ablation study of different module combinations on the SYUGV dataset.

Method	Input	P/%	R/%	mAP_0.5_/%	mAP_0.5–0.95_/%	Params/M	FPS
Baseline	VI + IR	93.17	88.04	94.06	68.94	8.8	163
Baseline+DBE	VI + IR	93.92	89.19	95.23	69.33	9.7	135
Baseline+CBAM	VI + IR	93.43	87.56	93.61	67.75	9.2	161
Baseline+ECA	VI + IR	93.28	89.01	94.50	68.72	17.6	98
Baseline+CTF	VI + IR	93.81	88.93	95.02	69.01	11.5	128
Baseline+DBE + TF	VI + IR	95.20	89.53	94.92	69.14	11.0	115
Baseline+DBE + CTF	VI + IR	95.26	90.48	95.64	69.63	12.3	117

**Table 7:** Ablation study of different module combinations on the LLVIP dataset.

Method	Input	P/%	R/%	mAP_0.5_/%	mAP_0.5–0.95_/%	Params/M	FPS
Baseline	VI + IR	93.52	87.01	93.98	57.61	8.8	155
Baseline+DBE	VI + IR	94.96	87.74	94.39	58.70	9.7	120
Baseline+CBAM	VI + IR	94.91	86.80	94.14	57.90	9.2	151
Baseline+ECA	VI + IR	94.20	87.49	94.85	59.33	17.6	92
Baseline+CTF	VI + IR	93.94	86.67	94.02	58.29	11.5	122
Baseline+DBE + TF	VI + IR	94.75	88.12	95.07	60.14	11.0	114
Baseline+DBE + CTF	VI + IR	95.39	89.51	95.70	61.67	12.3	113

The incorporation of only the DBE module improves the mAP@0.5 over the Baseline by 1.17% (from 94.06% to 95.23%) on SYUGV and 0.41% (from 93.98% to 94.39%) on LLVIP. This enhancement surpasses that achieved by applying other attention mechanisms like CBAM [43] or ECA [44] to the dual-branch network, demonstrating the specific effectiveness of our interactive enhancement design for bimodal features.

Adding only the CAT module results in a moderate improvement (+0.96% on SYUGV, + 0.04% on LLVIP), indicating that fusion alone provides limited gains without enhanced feature specificity from DBE.

The full model (Baseline+DBE + CAT) achieves the highest mAP@0.5 on both datasets (95.64% on SYUGV, 95.70% on LLVIP). The combined improvement (+1.58% on SYUGV, + 1.72% on LLVIP) is greater than the sum of individual gains, revealing a clear synergistic effect where the DBE-enhanced features enable more effective fusion via CAT.

2) Convergence and Stability

**Fig 15:**
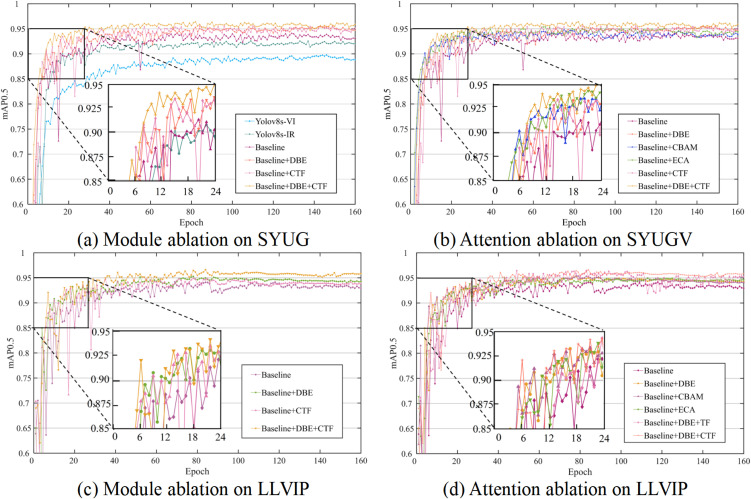
mAP@0.5 curves of ablation studies on the(a) SYUGV and (b) LLVIP datasets.

The mAP@0.5 curves during training are plotted in Fig 15. It is observed that the model combining both DBE and CAT converges faster and more stably to a higher performance plateau compared to all other variants, further confirming the robustness introduced by the proposed modules.

3) Visualization of Feature Focus

**Fig 16:**
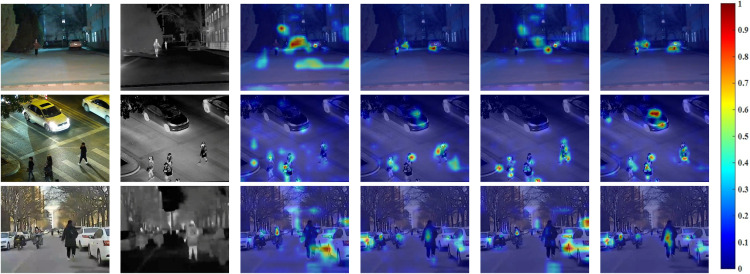
Visualization of feature activation (heatmaps) for different model variants.

The feature activation heatmaps for different model variants are displayed in Fig 16. It can be observed that: (1) The attention of the Baseline model is relatively dispersed; (2) The DBE module helps the model focus more precisely on the target objects themselves; (3) The CAT module alone cannot fully utilize complementary information without prior enhancement from DBE; (4) Only when DBE and CAT are combined does the model achieve precise and focused attention on the complementary features from both modalities, visually validating the mechanism of the proposed approach.

## 5 Conclusions

In this paper, a target detection method based on dual-modal fusion network DEF-Net is proposed. Firstly, a dual-branch feature encoder suitable for infrared and visible dual-input is constructed, and the dual-branch coding network is used to extract dual-modal features respectively. Secondly, in order to make better use of dual-modal features for object detection in complex scenes, the DBE module is proposed, which uses internal feature attention to enhance the information interaction between modalities. In addition, the cross-attention mechanism is introduced to further improve the expression ability of the network for complementary information, so as to effectively improve the detection performance of the model. Experimental results show that compared with the existing methods, the proposed method performs well on the SYUGV dataset. In the face of complex environments such as mutual occlusion of targets and loss of single-modal information caused by local transition exposure, the proposed method still maintains good detection performance and has strong robustness. Future work will increase the complexity of the scene and expand the dataset, and explore ways to improve the generalization ability of the model to make it more applicable to actual unmanned detection systems.

## Supporting information

S1 File Running the code: DEF-Net. (RAR)
